# Positive/Negative Temperature Coefficient Behaviors of Electron Beam-Irradiated Carbon Blacks-Loaded Polyethylene Nanocomposites

**DOI:** 10.1021/acsomega.2c05806

**Published:** 2022-12-09

**Authors:** Choong-Hee Kim, Seul-Yi Lee, Soo-Jin Park

**Affiliations:** † Department of Chemistry, Inha University, 100 Inharo, Incheon22212, Republic of Korea; ‡ KIURI Center for Hydrogen Based Next Generation Mechanical System, Incheon21999, Republic of Korea

## Abstract

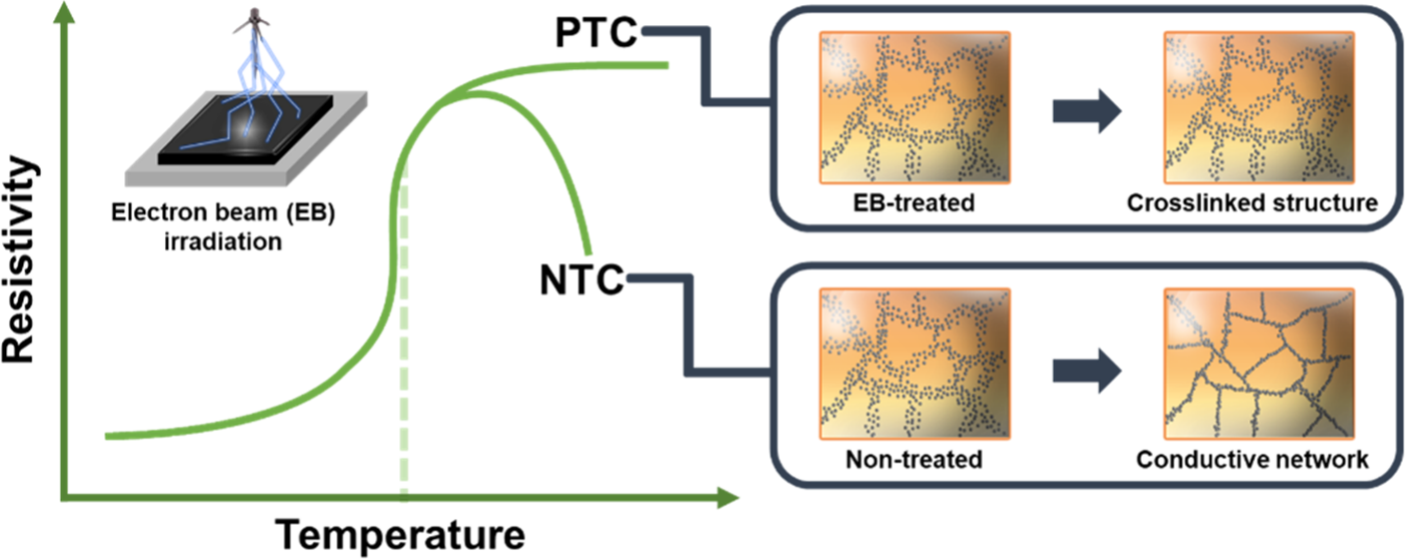

Polymer-based materials with positive temperature coefficients (PTC) are regarded as potential candidates for electrical heating elements in a wide range of applications, such as wearable electronics, soft robots, and smart skin. They offer many advantages over ceramic or metal oxide-based composites, including low resistance at room temperature, excellent flexibility and processability, and low cost. However, the electrical resistance instability and poor reproducibility have limited their use in practical applications. In this work, we prepared carbon blacks-reinforced high-density polyethylene nanocomposites (CBs–HDPE) loaded with polar additives (polyols or ionomers), which were subsequently subjected to electron beam (EB) irradiation to explore their PTC behaviors. We found that the EB-treated nanocomposites exhibited PTC behaviors, while the untreated samples showed negative temperature coefficients. Further, EB–ionomer-CBs–HDPE showed the highest PTC intensity of 3.01 Ω·cm, which was ∼35% higher than that of EB-CBs–HDPE. These results suggested that the EB irradiation enabled a specific volume expansion behavior via enhanced crosslinking among CBs, polar additives, and HDPE, inhibiting the formation of conductive networks in the nanocomposites. Thus, it can be concluded that polar additives and further EB irradiation played an important role in enhancing the PTC performances. We believe the findings provide crucial insight for designing carbon–polymer nanocomposites with PTC behaviors in various self-regulating heating devices.

## 1 Introduction

Conductive nanoparticle-loaded polymeric composites (CPCs) have attracted great interest from both academia and industry due to their wide applications, such as wearable electronics, soft robots, smart skin, and electromagnetic interference shielding.^1−4^ The electrical conductivity of the CPCs is influenced by the filler and loading fraction as well as preparation conditions, such as temperature.^5,6^ The contact between conductive fillers at the interface of insulating polymer particles facilitates the formation of efficient conductive paths, achieving enhanced electrical conductivity.^7,8^ A loading fraction of the filler, associated with the insulating–conducting transition, can be a key factor to forming an efficient conducting network between fillers, which affects the tunneling and hopping conduction. It is referred to as the percolation threshold in the composites.^9,10^ In addition, the crystallinity of the polymer can also be an important factor in forming the conducting paths in the CPCs. In particular, the formation of conductive paths can be caused by the decrease in the average interparticle or aggregate distance of the conductive fillers in a semicrystalline polymer. This can contribute to a larger thermal expansion upon the melting of the crystalline phase compared with the amorphous phase.^11^

Carbon blacks (CBs) are widely used in a variety of applications, such as conductive composites, batteries, fuel cells, and so forth.^12−15^ CBs are very well known for their very high filler loading contents by forming a percolating 3D network when the distance between aggregates is low. These are attributed to their specific structure with a low aspect ratio and a round shape in comparison with other conductive fillers, such as CNTs or graphene (which normally have a percolation threshold of a few % by volume).^16−18^ Also, CBs-loaded nanocomposites have been attracting increasing attention as positive temperature coefficient (PTC) materials, owing to the advantages such as lower resistivity at room temperature, easier fabrication, and lower costs than ceramics.^19−21^

PTC materials present a positive correlation between electrical conductivity and temperature, leading to an increase in electrical resistivity with increasing temperature. In contrast, a decrease in resistivity with increasing temperature results from negative temperature coefficient (NTC) effects.^22,23^ These materials are used in self-controlled heaters, current limiters, and over-current protectors.^24−26^ However, the poor electrical reproducibility and the NTC behaviors represent outstanding challenges that remain to be solved to improve their performance in the ambient-temperature applications.^27,28^

High-density polyethylene (HDPE) is a typical semicrystalline polymer, commonly used as a matrix for PTC materials.^29,30^ In CB-loaded HDPE nanocomposites, strong NTC effects are occasionally observed above the PTC transition, when a new network is formed by the thermally induced motion of the conducting particles in the matrix. In order to limit these undesirable effects, various methods have been proposed to eliminate the NTC behaviors of the nanocomposites.^31−33^ One of the methods for controlling the NTC behaviors is to increase the crosslinking of the HDPE matrix and thus limiting the mobility of CBs inside the matrix.^34,35^

Since electron beam (EB) irradiation technology has been developed for a variety of plastic applications, such as cable sheaths, formed plastics, the radial tire industry, and so forth.^36,37^ The main advantage of this technology is that it can alter the inherent chemical, structural, physical, and thermal properties of polymers, thereby providing lower cost and higher product purity.^38,39^ These changes mainly arise from variations in the crosslinking/crystallinity of the polymers and/or the interactions between filler–filler, filler–polymer, and polymer–polymer. There are various literature on CBs–HDPE composites using EB, plasma, and so forth to improve the crystallinity.^40^ However, there has been rarely reported, to the best of our knowledge, on the synergetic effects of polar additives and further EB irradiation on the polymeric nanocomposites for PTC performances.

In this study, we prepared CBs–HDPE nanocomposites loaded with polar additives (polyol or ionomer), which were subsequently subjected to EB irradiation to explore their PTC behaviors. The morphology and chemical surface properties of the nanocomposites loaded with different polar additives before and after EB irradiation were investigated by scanning electron microscopy (SEM) and Fourier-transform infrared (FT-IR) spectroscopy, respectively. Pyroresistivity measurements were performed to study the PTC/NTC behaviors of the nanocomposites. The experimental results showed that the EB treatment strongly influenced the crosslinking in the nanocomposites, which exhibited PTC behaviors above the melting point temperature of HDPE. The present study provides a promising strategy for designing polymeric nanocomposites loaded with carbonaceous materials, with potential application in various self-regulating heating devices.

## 2 Experimental Section

### 2.1 Materials

**Figure 1:**
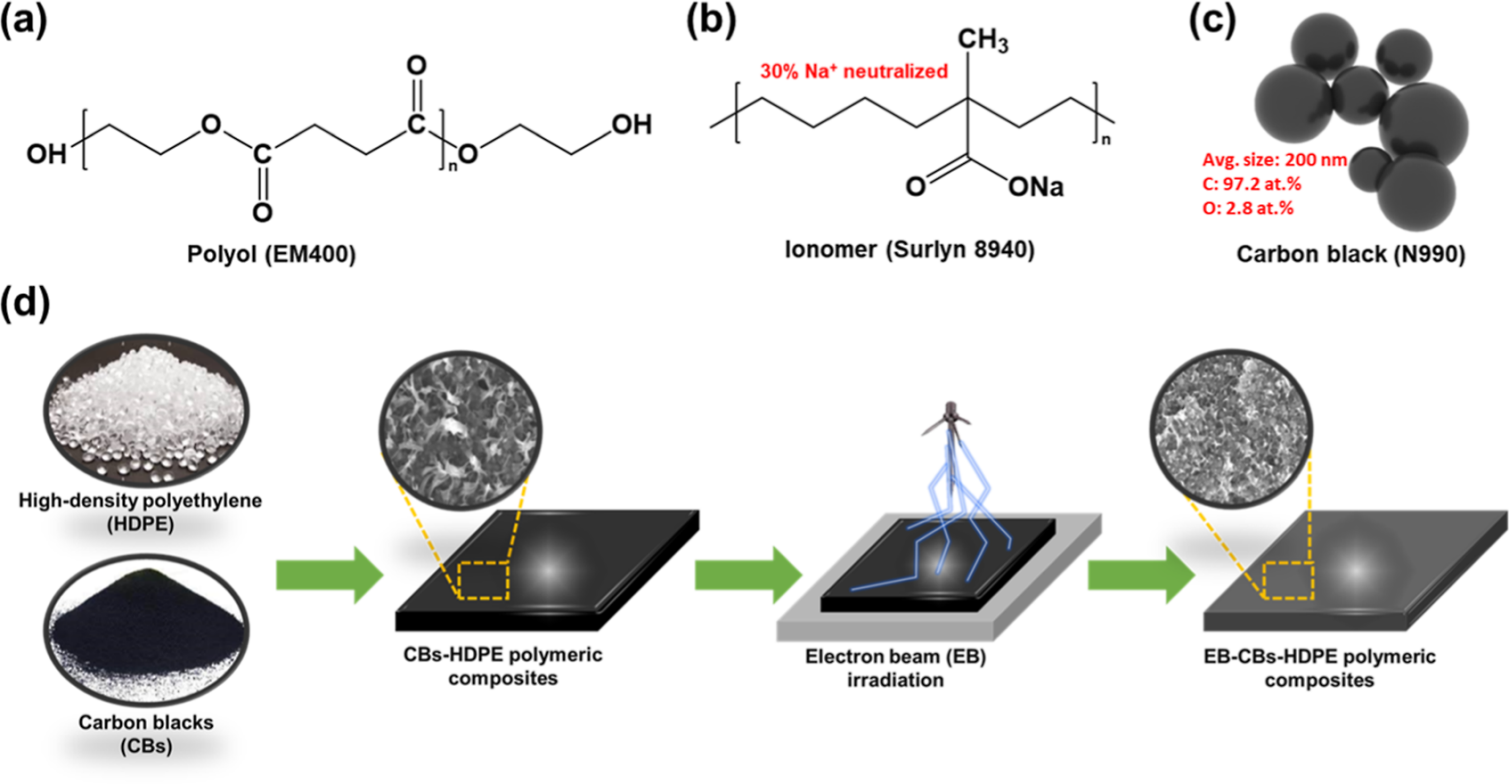
Chemical structures of (a) polyol (EM400), (b) ionomer (Surlyn 8940), and (c) CB (N990). (d) Schematic illustration of the fabrication process for EB-irradiated CBs–HDPE nanocomposites.

The polymer matrix consisted of HDPE (5200B, Hanwha Petroleum Chem Co., Korea) with a melting point of 126–136 °C and a density of 0.940–0.970 g/cm^3^. CB (N990, Korea Carbon Black Co., Korea) with a mean particle size of 200 nm and a specific surface area of 11 m^2^/g was used as an electrically conductive filler. EM400 (Honam Petrochemical Co., Korea) polyol and Surlyn 8940 (Du Pont Co., USA) ionomer were used as polar additives. The polyol is a polar resin compound containing multiple hydroxyl groups. The ionomer is a thermoplastic resin whose acid groups have been partially neutralized with sodium ions. Their chemical structures are displayed in Figure 1a–c.

### 2.2 Preparation of Composites

CB, HDPE, and polar additives were mixed by the conventional melt-mixing method at 50 rpm and 160 °C for 15 min. Compression molding was performed with 2 mm thick sheets using a hot press at 180 °C and 10 MPa. Table 1 lists the preparation compounding ratios of the samples. The same CB ratio was used for preparing all CBs–HDPE samples. The sample sizes used in the PTC/NTC measurements were 20 mm (diameter) and 2 mm (thickness). EB irradiation was performed using an ELV-4 instrument at a beam energy of 1 MeV and a belt speed of 2 m/min. The absorbed dose reached up to 250 kGy. The synthesis process and identification of the composite samples are illustrated in Figure 1d.

**Table 1:** Compound Formulation of CBs–HDPE Nanocomposites

samples	HDPE (%)	CBs (%)	polyol (%)	ionomer (%)
CBs–HDPE	35	65		
polyol-CBs–HDPE	30	65	5	
ionomer-CBs–HDPE	30	65		5

### 2.3 Characterization

The CB dispersion before and after mixing with HDPE was characterized by SEM (JEOL 840A) and FT-IR (Vertex80 V, Bruker) spectroscopy in the 3700–600 cm^–1^ range. The practical loading content of CBs was analyzed using a thermogravimetric analyzer (NETZSCH TG209 F3, ETZSCH, Selb, Germany). Differential scanning calorimetry (DSC) (DSC-6, PerkinElmer) was used to obtain the crystallinity, enthalpy of fusion, and melting point of the samples. The heating runs were from 30 to 300 °C at a rate of 10 °C/min under nitrogen conditions. The crystalline fraction (*X*_c_) was determined using the following formula
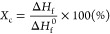
1where Δ*H*_f_ is the enthalpy of fusion (J g^–1^) determined from a DSC thermogram and Δ*H*_*f*_^0^ is the ideal enthalpy of fusion for HDPE (293 J g^–1^).

The heating electrode of the insulation resistance tester was connected to the controller. To measure the electrical resistivity, the manufactured nanocomposite materials were cut into circular pieces with a diameter of 2 cm and examined using a digital multimeter at an isothermal rate of 1 °C/min. The PTC properties of the prepared nanocomposites were measured using copper paste as a conductive binder. The PTC intensity (IPTC) was defined as the ratio of the maximum and room-temperature resistivities (ρ_max_ and ρ_RT_, respectively) calculated from the temperature dependence of the nanocomposite resistivities, as shown in eq 2.
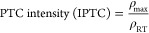
2

## 3 Results and Discussion

### 3.1 Morphology and Crystallization

**Figure 2:**
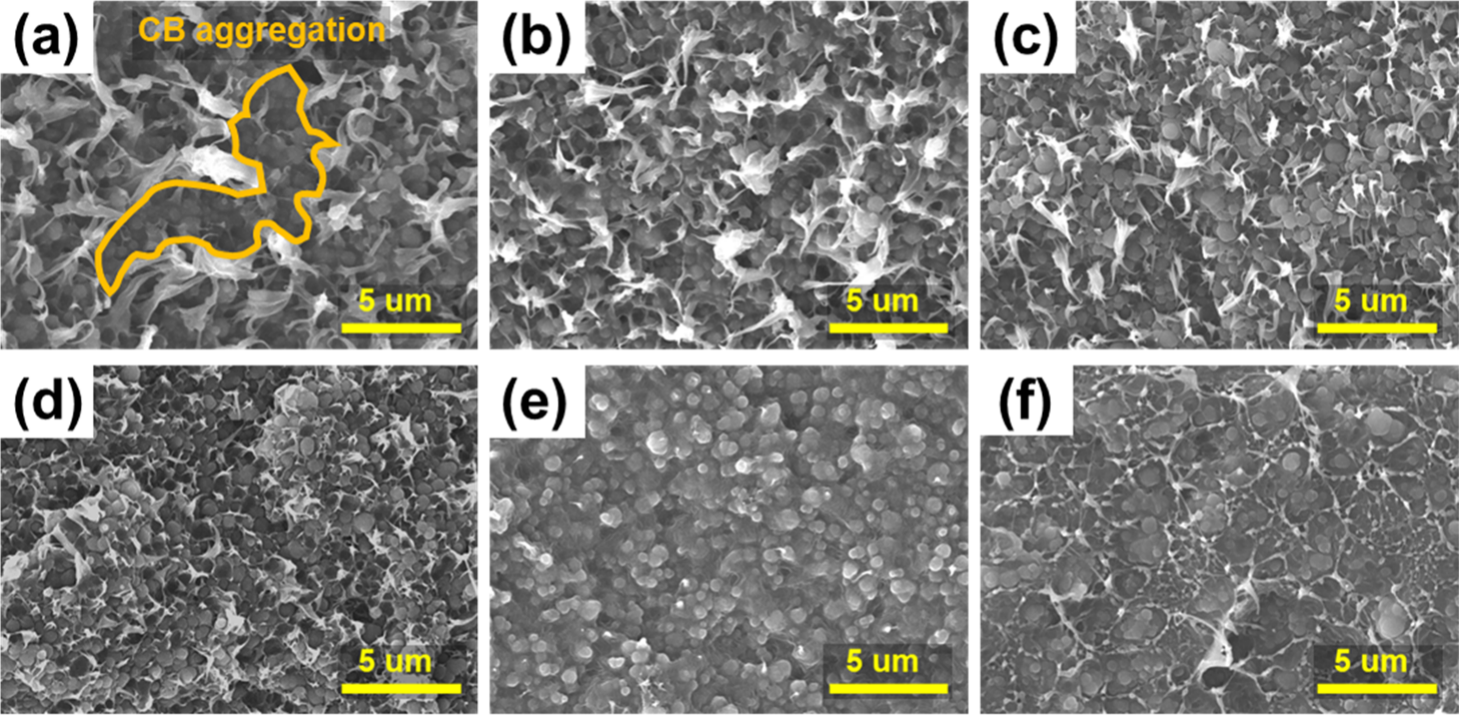
Comparative SEM images of CBs–HDPE nanocomposites for the effect of EB irradiation: (a) CBs–HDPE, (b) polyol-CBs–HDPE, (c) ionomer-CBs–HDPE, (d) EB-CBs–HDPE, (e) EB-polyol-CBs–HDPE, and (f) EB-ionomer-CBs–HDPE.

To investigate the dispersion state of the CBs, the morphologies of the CBs–HDPE nanocomposites before and after EB irradiation with different polar additives were observed by SEM. Figure 2a–c reveals agglomeration of CBs in the pure HDPE sample, whereas the nanocomposites loaded with polyol or ionomer polar additives were found to be more dispersed in the polymer matrix. This can be explained by the fact that the polar functional groups on the polar additive surface facilitate good interfacial bonding between the polar additives and CBs, enhancing the dispersibility of CBs in the HDPE matrix. The SEM images of the EB-CBs–HDPE nanocomposites in Figure 2d–f show a decreased agglomeration of the pure HDPE phase (white), compared to the untreated CBs–HDPE. The EB irradiation caused the cleavage of weak bonds in the polymer matrix and created new crosslinks between the CBs, polar additives, and HDPE.^39^ Given the system of the high content of CBs (65%) in our study, it can also be assumed that the HDPE permeates into the voids between CBs–CBs, enhancing the interfacial properties with CBs during the irradiation. These resulted in the phase reduction of the HDPE. Further, we found that the polar additives played an important role in improving the degree of dispersion of CBs in the nanocomposites, confirmed by the smoother surfaces in the polar additives-loaded samples compared to those of the unloaded samples. This phenomenon is similar to our previously published results.^34^

**Figure 3:**
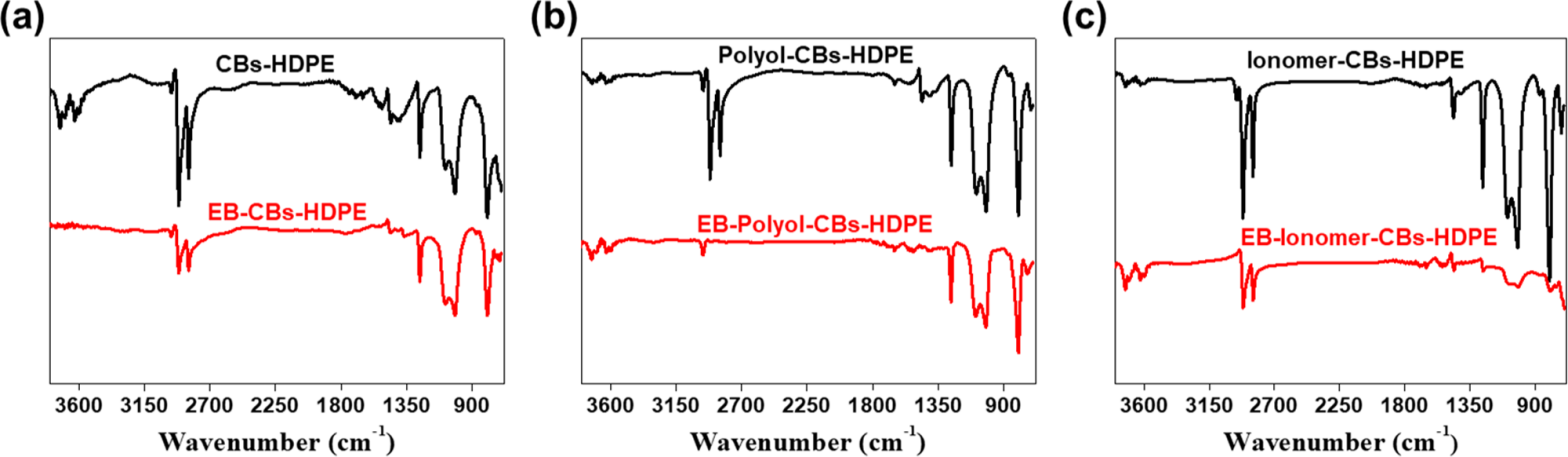
FT-IR spectra of CBs–HDPE nanocomposites before and after EB irradiation: (a) CBs–HDPE, (b) polyol-CBs–HDPE, and (c) ionomer-CBs–HDPE.

FT-IR analysis was performed to investigate the effect of EB irradiation on the functional groups of CBs–HDPE samples, as shown in Figure 3. Broadly speaking, the strong band in the range of 2830–2950 cm^–1^ is attributed to the sp^3^ aliphatic C–H stretching vibration of the −CH_2_ group. The FT-IR spectra of the CBs–HDPE nanocomposites before EB irradiation showed the prominent peaks centered at 2910 and 2850 cm^–1^, while the peaks diminished after EB irradiation. Furthermore, the peaks at 724, 1375, and 1463 cm^–1^ noticeably decreased after EB irradiation, which correspond to rocking deformation of the long-chain −CH_2_ group, bending of the −CH_2_ group, and symmetrical bending of the −CH_3_ group, respectively. These could be attributable to the decrease of the sp^3^ aliphatic carbons in the amorphous regions, while crosslinked structures emerged in the nanocomposites during the EB irradiation. With the introduction of polar additives, it was also found that the intensities were significantly decreased in the EB-polyol-CBs–HDPE and EB-ionomer-CBs–HDPE. It can be implied that the polar additives played an important role in forming the crosslinked structures in the nanocomposites. From our results, we believe that the crosslinking was accomplished via the radiation-induced collapse of the surface functional groups of the nanocomposites.^41,42^

**Figure 4:**
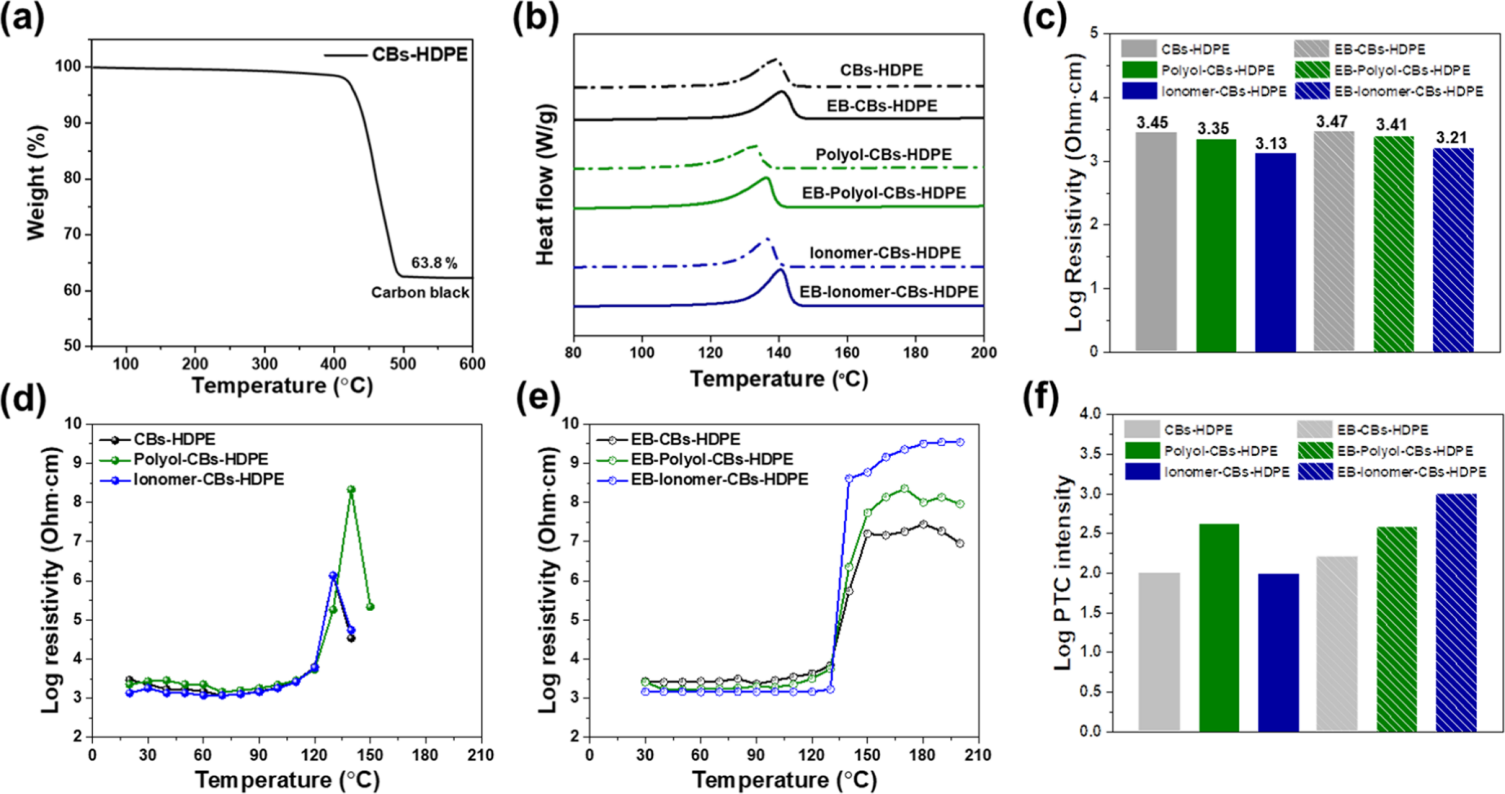
(a) TGA curves of CBs–HDPE nanocomposites, (b) DSC curves of CBs–HDPE nanocomposites, (c) logarithmic volume–resistivity of CBs–HDPE nanocomposites, (d,e) NTC/PTC behaviors of CBs–HDPE nanocomposites, that is, resistivities–temperature behaviors before and after EB treatments, and (f) PTC intensities before and after EB treatments.

We carried out thermogravimetric analysis (TGA) to confirm the practical loading content of CBs in the CBs–HDPE nanocomposites, as shown in Figure 4a. It exhibited that the thermal decomposition of the HDPE matrix occurred between 400 and 500 °C. After pyrolysis of HDPE, the weight of 63.8% above 500 °C remained, indicating the CB content in the composites.

DSC was performed to examine the effect of CBs and polar additives on the crystallization of CBs–HDPE nanocomposites before and after EB irradiation, as shown in Figure 4b. The melting temperature (*T*_m_), enthalpy of fusion (Δ*H*_f_), and crystalline fraction (*X*_c_) were determined as listed in Table 2. Normally, the volume expansion of a polymer, originating from the melting of the crystalline region and thermal expansion, is the key factor influencing the PTC effect of polymeric composites. A major peak was observed in all endothermic curves, corresponding to the melting points of the CBs–HDPE nanocomposites in the temperature range of 130–150 °C.^43^ The peaks shifted to slightly lower temperatures when the polyol or ionomer was incorporated in the nanocomposites, meaning the *T*_m_ decreased due to their inherent low *T*_m_ of the polar additives. By the same token, the decrease of *X*_c_ was also found in the polyol-CBs–HDPE and the ionomer-CBs–HDPE. It is well known that the crystallization of HDPE is governed by both crystal nucleation and growth. The nucleation sites are strongly influenced by the CB contents, which could play a significant role in the crystallinity.^44,45^ In our previously published study, we found that the CBs possessed unpaired radicals and active functional groups, which could react with the hydrogen atoms of HDPE. In addition, some branched chains of HDPE could react with the free radicals of the CBs, restricting their movement.^46^ After EB irradiation, the *X*_c_ of EB-CBs–HDPE was found to be 27.3%, which is an improvement of 9.2% compared to CBs–HDPE (bare sample). This indicates that the EB irradiation increased the degree of crosslinked structures in the CB–HDPE nanocomposites. The *X*_c_ values of EB-polyol-CBs–HDPE and EB-ionomer-CBs–HDPE showed 30.0 and 31.1%, which were increments of 20 and 24.4%, respectively, compared to CBs–HDPE. These data clearly demonstrate that the presence of polar additives and further EB irradiation led to the increase of the crystallinity in the resultant nanocomposites. Similarly, Narkis et al. reported that both EB irradiation and rubber additives as mechanical stabilizers had influence on further crosslinking of the polymer composites. They reported that the absence of the NTC effect in the crosslinked polymeric composites was closely related to an increase in the crystallinity of the polymer matrix. This is resulted from the reduction in the mobility of the CB particles in the composites.^47^

**Table 2:** Thermal Properties of the Samples Studied

samples	*T*_m_ (°C)	Δ*H*_m_(J g^–1^)	*X*_c_ (%)
CBs–HDPE	138.8	73.1	25.0
polyol-CBs–HDPE	132.8	67.0	22.9
ionomer-CBs–HDPE	136.4	67.1	23.0
EB-CBs–HDPE	140.6	80.4	27.3
EB-polyol-CBs–HDPE	136.2	87.7	30.0
EB-ionomer-CBs–HDPE	140.2	91.1	31.1

### 3.2 Pyroresistive Behaviors

Figure 4c shows the logarithmic volumetric resistivity of the CBs–HDPE nanocomposites loaded with polar additives before and after EB irradiation at room temperature. The electrical resistivity of all composites showed a slight decrease after adding the polar additives, indicating that a conductive pathway was generated. Our experimental results show that the polar additives promoted the dispersion of CBs during the preparation of the nanocomposites in the molten state. This is in good agreement with the SEM results (Figure 2). The unique structure of the polar additives with oxygen-containing functional groups suggests that they could act as dispersion agents.^48^ This might be enabled by the high loading of CBs (65 wt %) compared to the small traces of polar additives (5 wt %) in the present system, which facilitates the formation of the conductive networks.

Figure 4d–f shows the pyroresistive behaviors and PTC intensities of the CBs–HDPE nanocomposites with the polar additives before and after EB irradiation. As shown in Figure 4d,e, the logarithmic resistivity of the non-irradiated nanocomposites increased with increasing the temperatures and suddenly began to decrease near the melting point of HDPE (∼130 °C), which denoted an NTC effect. Moreover, the polar additives were found to play an important role in enhancing the NTC intensities. It is well known that the NTC effect can be attributed to the rearrangement and/or reformation of conductive pathways through the movements of CBs in the molten polymer matrix.^49,50^ On the other hand, a constant increase in the logarithmic resistivity of the EB-irradiated nanocomposites was observed at temperatures higher than 130 °C, revealing an intriguing PTC behavior. This effect can be ascribed to the abrupt volume expansion of the HDPE polymer matrix containing polar additives at the melting point temperature. This is because the crosslinked networks formed during EB irradiation played an important role in an effective way to reduce the freedom of movement of the CBs at high temperatures, thereby eliminating the NTC effect. These effects resulted in a random distribution of gaps between CBs in the nanocomposites, leading to the collapse of the conducting CB network. It is well documented that the PTC effects of CBs-loaded nanocomposites are mostly caused by an increased average distance between CBs and/or aggregates in the homogeneous polymeric matrix.^51−53^ In case of the CBs–HDPE nanocomposites with high CB content (65%) considered in this study, the CBs could easily generate a continuous conductive pathway, significantly reducing the interparticle distances. An increased interparticle distance might be the main factor causing an increase in resistivity. Thus, it is suggested that the EB irradiation influenced the crosslinking between HDPE and polar additives, suppressing the movements of the conductive fillers in the nanocomposite, which played a significant role in determining the PTC behaviors.^54,55^

Zhang et al. studied the PTC behaviors of EB-irradiated CBs–HDPE composites under different EB conditions, such as room and melting temperatures. They reported a higher degree of crosslinking for the composites formed by EB irradiation in the molten state, which strongly limited the mobility of CBs and macromolecular chains of HDPE, reducing the PTC intensities at room temperature. Nevertheless, it is suggested that the volume expansion of the polymeric matrix could induce a decrease in the volume fraction of CB’s agglomerate, resulting in the PTC effect.^56^ Jong et al. prepared the HDPE/CB composites with various manufacturing conditions. The EB-irradiated composites showed similar PTC behaviors, while the NTC effect was almost eliminated. They demonstrated that the crosslinking of the polymeric matrix reduces the movement of the CB particles at a higher temperature above the melting region of polymers; hence, resulting in the blocking of re-agglomeration of CB particles and PTC behaviors.^57^

**Figure 5:**
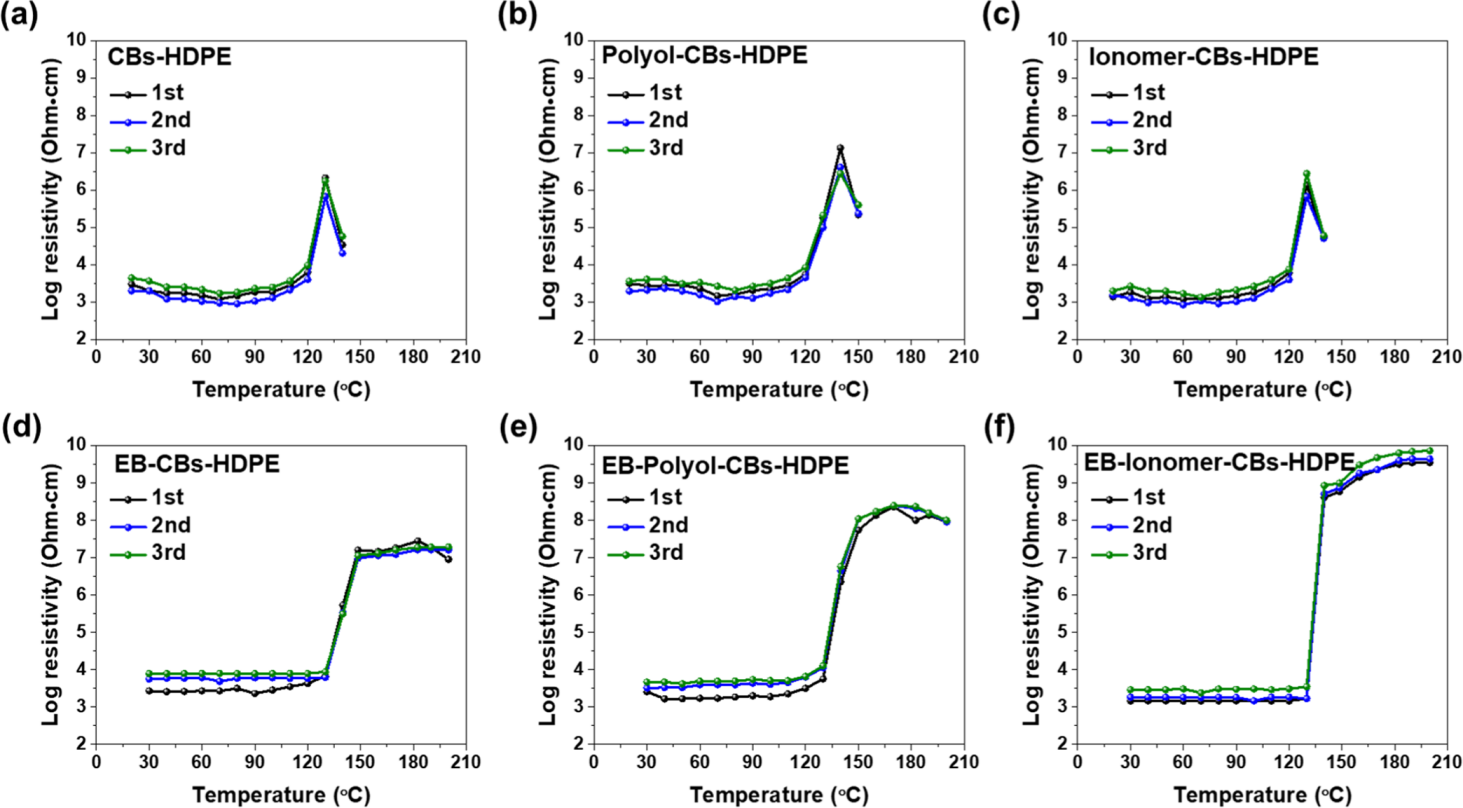
Reproducibility of pyroresistive behaviors of CBs–HDPE nanocomposites during the heating cycles: (a) CBs–HDPE, (b) polyol-CBs–HDPE, (c) ionomer-CBs–HDPE, (d) EB-CBs–HDPE, (e) EB-polyol-CBs–HDPE, and (f) EB-ionomer-CBs–HDPE.

Figure 5 shows the reproducibility of the pyroresistive properties of EB-CBs–HDPE nanocomposites. As expected, the EB-irradiated nanocomposites showed good reproducibility over three cycles. These results demonstrated that EB irradiation-induced crosslinking provides excellent electrical reproducibility and PTC properties, which can be attributed to the enhanced interactions between CBs and HDPE.^58^

**Figure 6:**
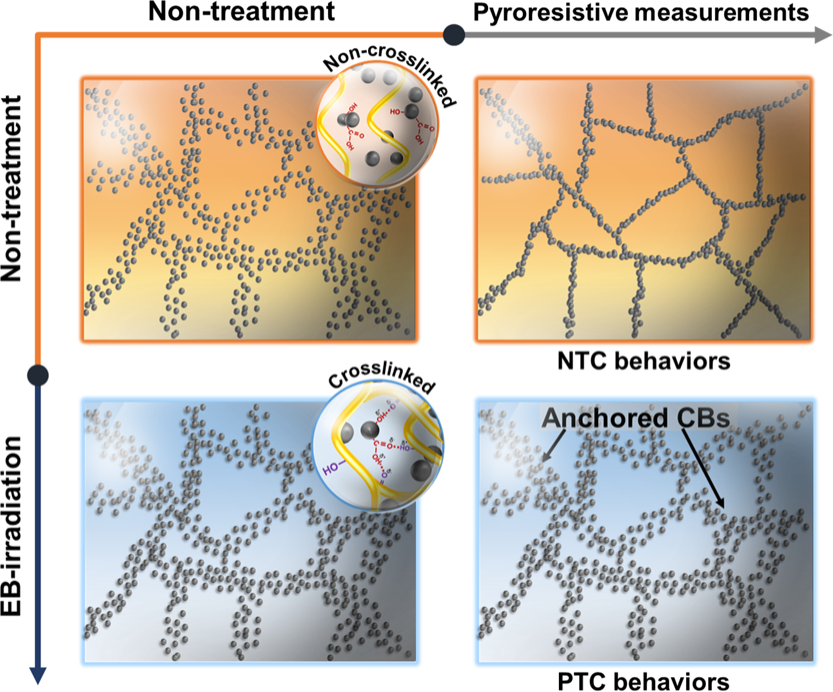
Schematic illustration of the movements of CB particles during pyroresistive measurements.

The different PTC/NTC behaviors of the CB–HDPE nanocomposites before and after EB irradiation are illustrated in Figure 6. For the EB-CB–HDPE nanocomposites, the EB irradiation generated crosslinked structures, enabling to efficiently anchor the CBs in the polymeric matrix. This prevented the formation of a conductive network near the melting point of HDPE (the PTC effect). On the other hand, an effective conductive network was formed in the untreated nanocomposites via the re-agglomeration of CBs, promoted by their relatively higher mobility and showing NTC behaviors.

## 4 Conclusions

We have investigated the effect of EB irradiation on the PTC/NTC performances of CBs-reinforced HDPE nanocomposites loaded with polar additives. The PTC/NTC behaviors were investigated by measuring their pyroresistive properties. The results showed that the EB-irradiated and untreated samples exhibited PTC and NTC behaviors, respectively. This suggested that EB irradiation led to an increased degree of crosslinking in the polymeric matrix, resulting in a specific volume expansion with increasing temperature and increasing the average distances between the CBs; this limited the formation of conductive networks in the nanocomposites. Hence, the PTC effect and electrical reproducibility were significantly improved by the higher crosslinked networks, which was attributable to the presence of polar additives and further EB treatments. Thus, it can be concluded that polar additives and further EB irradiation played an important role in enhancing the PTC performances. Based on the present experimental results, we believe that EB irradiation could be a promising strategy to obtain CBs-loaded HDPE nanocomposites with PTC behaviors.
